# Improving the quality of publications in and advancing the paradigms of clinical and social pharmacy practice research: the Granada Statements

**DOI:** 10.1136/ejhpharm-2023-003748

**Published:** 2023-03-13

**Authors:** Fernando Fernandez-Llimos, Shane Desselle, Derek Stewart, Victoria Garcia-Cardenas, Zaheer-Ud-Din Babar, Christine Bond, Ana Dago, Ramune Jacobsen, Lotte Stig Nørgaard, Carlo Polidori, Manuel Sanchez-Polo, Bernardo Santos-Ramos, Natalia G Shcherbakova, Fernanda S Tonin

**Affiliations:** 1 Revista Brasileira de Farmacia Hospitalar e Serviços de Saude, University of Porto, Porto, Portugal; 2 Research in Social and Administrative Pharmacy, Exploratory Research in Clinical and Social Pharmacy, Touro University California, Vallejo, California, USA; 3 International Journal of Clinical Pharmacy, Qatar University College of Pharmacy, Doha, Qatar; 4 Research in Social and Administrative Pharmacy, University of Technology Sydney, Broadway, New South Wales, Australia; 5 Journal of Pharmaceutical Policy and Practice, University of Huddersfield, Huddersfield, UK; 6 International Journal of Pharmacy Practice, University of Aberdeen, Aberdeen, UK; 7 Pharmaceutical Care España, Pharmaceutical Care España Foundation, Barcelona, Spain; 8 Exploratory Research in Clinical and Social Pharmacy, University of Copenhagen, Kobenhavn, Denmark; 9 Research in Social and Administrative Pharmacy, University of Copenhagen, Kobenhavn, Denmark; 10 Experimental medicine and Public health, University of Camerino, Camerino, Italy; 11 Ars Pharmaceutica, University of Granada, Granada, Spain; 12 Farmacia Hospitalaria, Hospital Universitario Virgen del Rocio, Sevilla, Spain; 13 Research in Social and Administrative Pharmacy, Western New England University, Springfield, Massachusetts, USA; 14 Pharmacy Practice, Instituto Politécnico de Lisboa, Lisboa, Portugal

## Abstract

Pharmacy and pharmaceutical sciences embrace a series of different disciplines. Pharmacy practice has been defined as ‘the scientific discipline that studies the different aspects of the practice of pharmacy and its impact on healthcare systems, medicine use, and patient care’. Thus, pharmacy practice studies embrace both clinical pharmacy and social pharmacy elements. Like any other scientific discipline, clinical and social pharmacy practice disseminates research findings using scientific journals. Clinical pharmacy and social pharmacy journal editors have a role in promoting the discipline by enhancing the quality of the articles published. As has occurred in other healthcare areas (ie, medicine and nursing), a group of clinical and social pharmacy practice journal editors gathered in Granada, Spain to discuss how journals could contribute to strengthening pharmacy practice as a discipline. The result of that meeting was compiled in these Granada Statements, which comprise 18 recommendations gathered into six topics: the appropriate use of terminology, impactful abstracts, the required peer reviews, journal scattering, more effective and wiser use of journal and article performance metrics, and authors’ selection of the most appropriate pharmacy practice journal to submit their work.

## Scientific fields and their achieving scientific paradigm

Disciplines are shaped by and in turn help to shape human behaviour.^1^ Several models developed over the past 50 years attempted to classify disciplines objectively. For example, Biglan and Becher, grounded in Lodahl and Gordon’s and Kuhn’s ideas,^2–4^ argued that fields with established paradigms (eg, physics, chemistry) have a high degree of consensus about theory, methods, and problems, while the opposite is observed for so-called ‘low-consensus’ disciplines such as humanities and the social sciences.^5^ According to the Recommendation Relating to the International Normalisation of Statistics on Science and Technology issued by the United Nations Educational, Scientific and Cultural Organization (UNESCO), fields of study or scientific disciplines broadly consist of: Exact and Natural Sciences, Engineering and Technology, Medical Sciences (including Pharmacy), Agricultural Sciences, Social Sciences, and Humanities. Yet, disciplines are not rigid, well-defined entities. Conversely, they are fluid, context-dependent and multi-scale phenomena built on repeated contributions (publications, academic works) and interactions (collaboration among researchers and other stakeholders).^1^ In this sense, it is even harder to describe, consistently define, and to attribute appropriate terminology to research areas where inter- and multidisciplinarity exist (reflecting different practices and interactions between disciplines), such as those within pharmacy. Traditionally, chemistry, biochemistry, physics, and physiology form pharmacy’s core knowledge base, but the social components (eg, humanistic, and social sciences) should also be recognised as a pillar of the practice of pharmacy.^6^

A lack of consistency and consensus attenuates a discipline’s progress and has a deleterious impact on its constituent scholars. Some of the findings from previous research indicate that scholars in low-consensus fields have a more difficult time publishing, tend to persist at ‘re-creating the wheel’, are less successful with acquisition of extramural grants, and have a poorer outlook on research and scholarship.^7^ This translates even to those scholars in university settings being less likely to be promoted in academic rank and even having lower salaries and poorer benefits than those who are in disciplines that have achieved greater scientific paradigm.^8^ The impact of research findings on professional practice and wider societal levels may be less in low-consensus fields.^9^

Clinical and social pharmacy practice are important research areas within the pharmaceutical sciences^9 10^ that have undergone (and are still undergoing) substantial changes. As what might be considered lower consensus fields, these two research areas are currently beset by a lack of agreement and a common understanding of what constitutes their very core, often being associated only with evaluating narrowly focused pharmacy services.^6 11^ Although no universally accepted definition for pharmacy practice research exists, the International Pharmaceutical Federation Pharmacy Practice Special Interest Group (FIP PPR-SIG) defined it as ‘the scientific discipline that studies the different aspects of the practice of pharmacy and its impact on healthcare systems, medicine use, and patient care’.^12^ A common misinterpretation of the nature of this field is confounding the term ‘practice’ with ‘practical issues’ and ignoring the theoretical bases that ultimately will support clinical and social pharmacy interventions. Kerlinger and Lee point out that the aim of science is theory; and theory is ‘a set of interrelated constructs, definitions, and propositions that present a systematic view of phenomena by specifying relations among variables, with the purpose of explaining and predicting the phenomena’.^13^ Furthermore, clinical pharmacy aims to optimise the utilisation of medicines through practice and research in order to achieve person-centred and public health goals.^14^

The scope of pharmacy practice has expanded over the past decades to encompass clinical, behavioural, economic, and humanistic implications of the practice of pharmacy, as well as the implementation of innovations in practice (eg, health interventions, patient-care services), which are often provided in collaboration with other healthcare professionals (eg, physicians, nurses).^12 15^ Thus, it may not be easy to identify clinical and social pharmacy practice as basic research within an applied research discipline. Both types of research produce ‘new knowledge’, with basic research disciplines creating ‘knowledge of the underlying foundations of phenomena and observable facts’, while for applied research disciplines the knowledge created is ‘directed primarily towards a specific, practical aim or objective’.^16^ Clinical and social pharmacy practice researchers do both.

Publication patterns and practices are one of the differential characteristics of a scientific discipline. Publishing refereed work is a hallmark of science, primarily aiming at disseminating new, advanced, and high-quality research knowledge and findings as widely as possible in a timely and efficient manner. Regardless of the scientific publishing mechanisms—which have significantly evolved over the years, especially in response to technological progress^1 17^—this practice traverses all different academic or scientific disciplines, but customs and habits (eg, paper length and structure, title details, citation patterns) are different across disciplines. The aforementioned on scientific progress would indicate a need for a discipline’s journals, its authors, reviewers, and even its readers/followers to come together on important aspects that help propel its scientific paradigm.^7 18^

With the aim to identify the elements that may reinforce clinical and social pharmacy practice as a scientific discipline by consolidating common publication patterns, a group of pharmacy practice journal editors met in June 2022 in Granada, Spain. As a consequence of this meeting, a series of recommendations to improve publication patterns in pharmacy practice was created, that is, these ‘Granada Statements’. This type of initiative is not unprecedented. In 1978, a group of medical journal editors gathered in Vancouver, Canada to create the ‘Uniform requirements to submit a paper to a medical journal’. Years later, this group became the International Committee of Medical Journal Editors (ICMJE - https://www.icmje.org/), which is now one of the most used standards in scholarly publishing. A similar initiative was created approximately 30 years ago for nursing with the International Academy of Nursing Editors (INANE - https://nursingeditors.com/).

With this paper, which will be simultaneously published in several clinical and social pharmacy practice journals, the Pharmacy Practice Journal Editors Group offers the Granada Statements as a set of recommendations for pharmacy practice authors, reviewers, and journal editors aiming to strengthen pharmacy practice as a discipline. The Granada Statements comprise 18 recommendations grouped in six topics: the appropriate use of terminology, impactful abstracts, the required peer reviews, journal scattering, more effective and wiser use of journal and article performance metrics, and authors’ selection of the most appropriate pharmacy practice journal to submit their work.

## The appropriate use of terminology in publishing

One of the differential characteristics of disciplines with a high degree of consensus is the consistent use of precise terms to refer to each concept. Several areas have created task forces to maintain glossaries. The International Union of Pure and Applied Chemistry (https://iupac.org/) and the International Union of Basic and Clinical Pharmacology (https://www.guidetopharmacology.org/) are good examples of this procedure.

Clinical and social pharmacy practice have been accused of inconsistent terminology use, whether in journal titles or in articles.^19 20^ This inconsistent terminology use is evident in the lack of a common branding: clinical pharmacy, pharmacy practice, social pharmacy, administrative pharmacy. This confusion is even greater when considering the terminology used to describe pharmacists’ interventions or services: medicines management, polypharmacy management, pharmaceutical care, medication therapy management, comprehensive medication management, etc.^1 21^ One could argue that slight differences exist among these terms. However, several consequences emerge when using many different terms for slightly different concepts, which were probably insufficiently defined.^22^ A first consequence is the existence of a variety of terms that should be used in search strategies of evidence-gathering exercises such as systematic reviews, which renders them not so systematic, after all.^23^ The final goal of a systematic review is to support evidence-based policymaking. A systematic review that insufficiently compiles the evidence about a topic may lead to inappropriate policy decisions. But perhaps the most harmful consequence for the visibility and relevance of the clinical and social pharmacy practice field is the invisibility of many articles resulting from their inability to be retrieved from bibliographic databases.^24^

One might think that subject headings (eg, Medical Subject Headings—MeSH) were created to classify articles and are especially important when authors do not use standardised terminology. MeSH terms have been known in pharmacy since their inception.^25^ Unfortunately, clinical and social pharmacy practice were highlighted as a field where MeSH use is scarce in comparison with other areas.^26^ It is important to keep in mind that new MeSH terms can be suggested to the National Library of Medicine (NLM), but MeSH staff will only consider MeSH that correspond to terms frequently used in the literature.^27^

**Granada Statements**

1. Clinical and social pharmacy practice researchers should establish a commonly accepted glossary and use terms in a consistent manner.

2. Pharmacy practice and social pharmacy reviewers and journal editors should ensure standardised terminology is used in the articles they review and publish.

## Impactful abstracts

In addition to the reduced number of MeSH terms defining clinical and social pharmacy practice elements, a poor allocation of existing MeSH to pharmacy practice articles has been reported.^28 29^Also, an excessive indexing delay (ie, MeSH allocation) was observed for pharmacy articles.^30 31^ MeSH terms are crucial to ensure a more efficient literature retrieval, which will result in a higher visibility of the article and subsequently of the field. The role MeSH plays in a systematic search is not substituted by the author-listed keywords commonly used by journals. These keywords are not indexed in the abstract field of bibliographic databases and, although some databases have specific fields for them (ie, PubMed’s OT—Other Terms), they are only retrieved as abstract words (no additional benefit to use these words as keywords).

In the recent past, allocation of MeSH terms to articles indexed in MEDLINE was a responsibility of NLM cataloguers. Since the NLM announcement of the complete implementation of the Medical Text Indexer First Line indexing (MTIFL) that will select the MeSH, authors, reviewers and journal editors should take responsibility for the appropriate allocation of MeSH terms to the articles.

MTIFL is an automated natural language processing system which identifies the appropriate MeSH terms from the MeSH thesaurus using only the text in article title and abstract. As stated by the NLM, after mid-2022, all articles indexed in MEDLINE will have MeSH terms allocated by MTIFL, mechanistically rather than through human judgement/intervention. This modification of the process increases even more the relevance of the title and abstract, that in the past had a role only in summarising the content of the article and helping potential readers to decide proceeding to the full text article.

The MTIFL system tries to match words and n-grams included in the title and the abstract not only with the MeSH term (ie, descriptor), but also with the other ‘concept terms’ associated with the descriptor, which can be easily identified as ‘entry terms’ in the MeSH database (https://www.ncbi.nlm.nih.gov/mesh/). Thus, if an article’s title or abstract includes the exact wording of any of these descriptors or entry terms, the system will allocate the given MeSH to that article.^20^

**Granada Statements**

3. Clinical and social pharmacy practice researchers should use existing MeSH (Medical Subject Headings) terms as part of their titles and abstracts.

4. Clinical and social pharmacy practice reviewers and journal editors should ensure that authors included the most appropriate MeSH terms in the articles they review and publish.

## The required peer reviews

Since the 18th century,^32^ scholarly publishing has been based on the contribution of colleagues in assessing and improving the original text submitted by the authors by means of the peer review process.^33^ Based on Linus’s law (ie, ‘given enough eyeballs, all bugs are shallow’), the rationale of peer review is to avoid errors^34^ and to increase the quality of publications.^35^ Although peer review has been strongly criticised^36^ and systematic reviews could not demonstrate the added value of this process,^37 38^ more reliable alternative systems do not exist.^39^ Pre-prints with post-publication review have been proposed as a solution to have scientific publications more rapidly accessible. Many forces, mainly outside the research workforce, are insisting on the benefits of publishing findings in a preprint server and waiting for future comments, but in-depth analyses of the consequences of this practice have not been undertaken. The scientific community, and not external influencers, should decide if the scholarly publication system should move into a social media publication system, or if pre-publication peer review is a prerequisite. This is an urgent decision because all the participants in the publication process might appear to be unhappy:

- Authors tend to complain about peer review for several reasons (ie, excessive reviewers’ criticism^40^), but the most common complaint is related to the duration of the publication process.^41^ However, studies have demonstrated that the time to get a manuscript accepted in biomedical journals is about 100 days, and clinical and social pharmacy practice journals do not substantially differ.^42^
- Editors tend to complain about the difficulty of identifying at least two reviewers to accept the task of reviewing each manuscript^43^ and about the timeliness and quality of the reviewers’ comments. Although shortage of reviewers is affecting journal operations and practices, editors should keep in mind that the workload of reviewing articles can be onerous for individuals and institutions^44^ and that reviewers provide the service altruistically.^45^
- Reviewers tend to complain about the excessive number of peer review requests they receive. But they should consider that the number of review invitations they receive depends only on the number of reviewers requested for each manuscript and the journal’s rejection rate.^46^ Editors can reduce the number of review requests by considering desk rejection rates (ie, rejection without external peer review) of papers unlikely to be accepted by reviewers, even if that is not the most favourable outcome to most authors, even while doing so expeditiously helps authors ‘move on’.^47^

It is important to understand that these three participants (ie, authors, reviewers and editors) are in fact only one group of researchers acting in three different roles at different points in time.^48^

**Granada Statements**

5. Clinical and social pharmacy practice researchers should be more proactive in becoming involved as peer reviewers to reduce the duration of the publication processes.

6. Clinical and social pharmacy practice educators and supervisors should mentor their students to serve as peer reviewers.

7. Clinical and social pharmacy practice journal editors should carefully find a balance between the number of manuscripts they submit to external peer review and those that are desk rejected.

8. Clinical and social pharmacy practice journal editors and publishers should consider systems to reward peer reviewers’ efforts, including public recognition of their contribution at an article level.

9. Clinical and social pharmacy practice peer reviewers should be reminded that their highly valuable role improves the quality of the manuscripts; hence it is incumbent upon them to provide constructive, quality reviews within the given timeframe.

## Journal scattering

Studies have demonstrated that pharmacy practice authors tend to scatter their articles among a huge number of journals outside the area.^28 29^ It is often argued that this dispersion enhances the visibility of findings for the authors and for the discipline. With more than one million articles published in biomedical journals each year, one should accept that bibliographic databases are the correct way of accessing articles published. The prior alternative of paying attention to a limited number of tables of contents is insufficient and may bias or attenuate the knowledge gained. Researchers can hardly complain about limited exposure and impact of journals in the discipline when they submit and publish their ‘best work’ outside of it.

Despite the existence of some meta-journals (ie, journals without a clear scope), most journals have not only a precisely defined scope, but also publication priorities. For example, in clinical and social pharmacy practice, some journals are interested in a more clinical approach, while others prefer more methodological papers, or social aspects of the practice. And for sure, any of these journals has a deeper knowledge in clinical and social pharmacy practice than any journal from other scientific areas.

To ensure the effectiveness of the peer review process, reviewers should have a deep knowledge of the concepts and the recent advances in clinical and social pharmacy practice. These colleague reviewers, together with the editor-in-chief and the associate editors, possess a deep knowledge of the area and the topic of the manuscript submitted, which should result in more constructive comments that will improve the paper. These persons should also be responsible for ensuring the use of consistent terminology and that the abstracts contain the terms that will be mapped into the appropriate MeSH terms.

**Granada Statements**

10. Clinical and social pharmacy practice researchers should prioritise pharmacy practice and social pharmacy journals for some of their ‘best’ papers and work to ensure the quality of the publication process considering the specific details of the area, even while seeking wider audiences as appropriate for various components of their work.

11. Clinical and social pharmacy practice educators and supervisors should promote pharmacy practice journal centredness among their students.

12. Clinical and social pharmacy practice journal editors should give priority to clinical and social pharmacy practice articles.

## Using the metrics wisely

One of the hidden reasons why researchers tend to publish their pharmacy practice articles outside of pharmacy practice journals may be the search for higher impact metrics. Inappropriate researchers’ performance assessment processes converted the ‘publish or perish’ into an ‘aim high’ obsessive goal for authors.^49^

Among several bibliometric indexes, impact metrics, such as the Impact Factor Score, have achieved an overwhelming position or level of currency in discussing the weight or gravitas of journals.^50^ Journal-based impact metrics have been criticised for several conceptual errors in the formulae,^51^ for poor transparency in their calculation,^52 53^ but more importantly for their relative inability to ascribe quality to papers published in these journals.^54–56^ Recognition of these issues led to the San Francisco Declaration on Research Assessment (https://sfdora.org/), which issued a plea to avoid use of journal-based metrics for the assessment of individual authors’ quality of papers and scientific prowess and productivity. Alternatives to journal-based metrics exist, that is, individual-based metrics, which might sometimes be more useful to evaluate the impact of a stream of scholarship, if not the contribution of individual papers.^57^ The European Commission has signed the Agreement on Reforming Research Assessment, which discusses moving away from use of metrics like the Impact Factor Score in evaluating quality of a scientific contribution.^58^

Notably, impact metrics have often underrated the scientific contribution of papers in the clinical and social pharmacy practice areas.^59^ They provide low coverage of many journals in the databases used to extract citations and often lack any semblance of a pharmacy practice subject category,^9 10^ often including pharmacy practice journals under Pharmacology and Pharmacy,^60^ thus placing papers from our discipline into a category with high-consensus bench or biological sciences where higher citations are the norm.

Biomedical researchers and some librarians^61^ may not be sufficiently aware about the methods to compute these impact metrics. It would be important to demystify the role of these metrics, whether journal-based or individual-based, and to clarify among researchers what the role is of their articles and the references they have in the metrics calculations.

**Granada Statements**

13. Clinical and social pharmacy practice researchers should promote among their institutions the use of individual-based metrics to assess the performance of individuals.

14. Clinical and social pharmacy practice researchers, while maintaining autonomy, should be aware of the importance of the references they include in their published papers and consider the need to strengthen the discipline and its component journals in their manuscript bibliographies.

15. Clinical and social pharmacy practice educators and supervisors should educate undergraduate and postgraduate students in the responsible use of metrics.

16. Stakeholders in clinical and social pharmacy practice should consider broader bases rather than only journal-based metrics to connote quality and achievement in the disciplines.

## Selecting the most appropriate pharmacy practice journal

Pharmacy practice and social pharmacy, themselves, are composed of a broad swath of topics. Among the signatories to the Granada Statements several different scopes or foci can be found, including but not limited to: clinical, methodological, political, social, economic, educational, behavioural, hospital-based and community-based, practitioner considerations, patient considerations, pharmacoepidemiological issues, and many others. Submitting a clinical article to a methodologically-oriented journal, or vice versa, may lead to an immediate desk rejection, regardless of the quality of the manuscript.

Similar to what happens with journals from other health areas, pharmacy practice journals have not only their preferences and interests, but also editorial board members with deep knowledge in specific sub-areas of pharmacy practice.

**Granada Statements**

17. Clinical and social pharmacy practice journal editors should work with authors to identify the most appropriate journal to submit their scholarly work early in the process (ie, during and even before submission, if possible).

18. Clinical and social pharmacy practice authors should heed advice and direction coming from journal editors, editorial boards, and reviewers to not only improve the quality of the original manuscript, but also be positively inclined toward the recommendations given rather than create unnecessary acrimony among scholars in the discipline.

## The Granada Group journals’ joint description

The journals comprising the Granada Group producing these Statements stand in unison in their endeavour to promote the quality and status of research in clinical and social pharmacy practice, as well as to advance the scientific paradigm of the discipline and broaden the impact of our respective journals to an international audience within and outside of pharmacy. The journals recognise that they are part of a larger phenomenon in health services research, having much in common with journals outside of pharmacy practice, per se, yet focusing on some aspect of the medication use process. In light of the Statements offered here and in recognition of the need for the journals to recognise their commonality, assist authors with selecting the most appropriate venue to publish their work, and unite in their mission to promote all journals in the area, the Granada Group journals have agreed to a common introductory description among all. The shared description among all the Granada Group journals will then be followed by specific descriptions that then help to establish the unique niches and processes associated with each of them. The common introductory description used for all Granada Group journals is as follows.

The *European Journal of Hospital Pharmacy* is one of several journals in comportment with the Granada Statements publishing high-quality, peer-reviewed content in health services research specifically as it relates to some aspect of the medication use process. The medication use process includes but is not limited to the prescribing, preparation, dispensing, administration, adherence to, evaluation, monitoring, and outcomes associated with legend or with over-the-counter medications, incorporating the concept of clinical pharmacy which aims to optimise utilisation of medicines to achieve person-centred and public health goals. The medication use process includes attitudes, perspectives, knowledge, and behaviours of any actor in this process, including prescribers, pharmacists, pharmacy personnel, other health practitioners, patients, and caregivers. As such, the Granada Group journals often refer to ‘pharmacy’ in their title or description, as these persons are central to the medication use process; however, research articles, reviews, and commentaries can refer to any person involved in this process, as well as any evaluation (eg, pharmacoepìdemiological) of the drug products themselves or systems employed to optimise the use process.

The Granada Group journals share certain commonalities and also goals to improve the medication use process and the outcomes emanating from this endeavour; however, each journal has an established niche and is optimally suited for certain types of manuscripts. Further description of the aims and scopes of this journal can be found on the journal’s website at www.ejhp.bmj.com

## Summary

The Granada Statements were created with the strong conviction that pharmacy practice is a scientific discipline that deserves reaching the high-consensus discipline category. The recommendations in these Statements aim to contribute to increase the quality of the articles that pharmacy practice researchers publish to disseminate their scientific contributions. At the end of the day, a scientific area and the profession behind it will benefit from the advancements published in these articles. The advancement of pharmacy practice is a conjoint responsibility between pharmacy practice researchers, peer reviewers, editors, and publishers, where scientific articles should be seen as the means to disseminate new knowledge that will improve practice.

## Data Availability

No data are available.
